# Preoperative Planning Framework for Robot-Assisted Dental Implant Surgery: Finite-Parameter Surrogate Model and Optimization of Instrument Placement

**DOI:** 10.3390/bioengineering10080952

**Published:** 2023-08-10

**Authors:** Yan Wang, Wei Wang, Yueri Cai, Qiming Zhao, Yuyang Wang

**Affiliations:** School of Mechanical Engineering and Automation, Beihang University, Beijing 100191, China; by2107029@buaa.edu.cn (Y.W.); wangweilab@buaa.edu.cn (W.W.); mangozhao@buaa.edu.cn (Q.Z.); chinchilla@buaa.edu.cn (Y.W.)

**Keywords:** dental implant surgery, surrogate model, virtual fixture, particle swarm optimization

## Abstract

For robot-assisted dental implant surgery, it is necessary to feed the instrument into a specified position to perform surgery. To improve safety and efficiency, a preoperative planning framework, including a finite-parameter surrogate model (FPSM) and an automatic instrument-placement method, is proposed in this paper. This framework is implemented via two-stage optimization. In the first stage, a group of closed curves in polar coordinates is used to represent the oral cavity. By optimizing a finite number of parameters for these curves, the oral structure is simplified to form the FPSM. In the second stage, the FPSM serves as a fast safety estimator with which the target position/orientation of the instrument for the feeding motion is automatically determined through particle swarm optimization (PSO). The optimized feeding target can be used to generate a virtual fixture (VF) to avoid undesired operations and to lower the risk of collision. This proposed framework has the advantages of being safe, fast, and accurate, overcoming the computational burden and insufficient real-time performance of complex 3D models. The framework has been developed and tested, preliminarily verifying its feasibility, efficiency, and effectiveness.

## 1. Introduction

### 1.1. Background and Task

Tooth loss is one of the most common oral diseases, especially among middle-aged and elderly people [1,2,3]. Tooth loss not only reduces life quality, but is also related to other disorders [4,5]. Dental implant surgery, as one of the most powerful therapies [6], can restore the function of teeth [7,8]. However, traditional dental implant surgery mainly relies on manual operation by the surgeon, bringing more burden and a relatively lower accuracy. Additionally, in many regions where medical resources are scarce, many patients with tooth loss will not receive qualified medical treatment in time.

With the development of automation technology, robot-assisted dental implant surgery provides new solutions [9], with great potential to provide better surgery, free dentists from heavy work intensity, and alleviate the shortage of professional dentists in less-developed regions [10]. Given these advantages, robot-assisted dental implant surgery is likely to gradually become mainstream in the future [11].

This paper focuses on a new dental implant robot system (DIRS). To perform the surgery, it is necessary to feed the instrument into the specified position inside the oral cavity. For this task, our team has already proposed a virtual fixture (VF) for oral surgery in previous work [12], in which its geometry and effect mode was designed. However, in our previous work, the target should be defined manually, causing some uncertainty and risk of collision. Also, manual definition can increase time expenses. Therefore, our previous work is incomplete; an automatic preoperative placement method, which can define the target and VF without any participation by surgeons, is needed to expand the previously proposed VF method to finish the task safely and efficiently.

### 1.2. Related Works

In recent years, robotics for oral and dental surgery has been studied. Sun et al. developed an implant robot system by introducing coordinate measurement equipment [13,14]. Yu et al. developed an image-based implant system. In their work, the artificial potential field method was adopted for navigation [15]. From 2018 to 2020, a research team from the University of Hong Kong designed a cable-driven robotic system for dental surgery [16], built master-slave mapping, analyzed system stiffness [17], and proposed a compensation strategy for the system [18]. Later, in 2021, researchers from Shanghai Jiaotong University developed a dental implant system based on a hybrid serial-parallel mechanism, with a real-time iterative trajectory-generating algorithm proposed [19]. The system is also combined with a force-based dragging control strategy and a 3D navigating system [20]. Additionally, a trans-oral robot was developed for COVID-19 PCR testing [21]. Hence, comprehensive works have been conducted for developing robot-assisted oral surgery systems, making contributions to the emerging field.

Since most surgical robots are designed in the form of master-slave control or teleoperation, to perform safe guidance, VF is a key issue, including its definition, generation, and application. The concept of VF was first proposed by Rosenberg [22]. Then, the main framework of virtual fixture was established by defining different types of VFs [23,24], and the admittance control strategy was combined [25,26]. Subsequently, some application strategies were proposed by scholars. Tang et al. replaced the organs with a set of bounding boxes, which were preoperatively defined through 3D measurement, and then established the VF through collision detection and force feedback [27]. Their method can improve safety during master-slave operations. Also, some novel geometry-based VF for robot-assisted surgery were proposed, including the offset surface of CAD models [28], or training a neural network to fit the geometry of the organ to build a virtual fixture [29]. Furthermore, force feedback control can be applied to VF. A team from Johns Hopkins University established a VF by converting the contact force to the adjusting velocity of the instrument for a leader-follower dual-arm surgical robot [30]. The above works include the basic framework and different application strategies of VF, and are very helpful for the work in this paper.

In terms of preoperative positioning and planning, although it is usually not the most critical issue, it is still useful for safe operation. Some researchers were attracted and developed some methods for preoperative positioning and planning. Yu et al. proposed an automatic preoperative positioning method based on parameterization and reinforcement learning [31] which built the model of the lesion and its spatial relationship with the surgical robot, obtaining the best position and direction to intervene in the body and where to place the surgical robot. A research team from Tianjin University assessed the motion skill and kinematics of a surgical robot [32,33], which was used to optimize preoperative planning. Preda et al. developed a preoperative planning software called iMTECH, which can determine the optimal position for surgery [34]. Badani et al. optimized the placement of the camera port for invasive surgery to minimize the risk of collision [35]. Banez et al. conducted similar works to determine an optimal port placement [36]. In a word, for preoperative positioning, safety, dexterity, and the workspace are typical items to be considered, and optimization methods are often adopted to obtain a desirable setup before the surgery.

Except for classic models or methods, some AI-based modern approaches can also make sense in the field of medical robotics, especially for detecting [37,38], sensing [39], and planning, which can provide a broader spectrum for dental implanting tasks. For example, by exploiting the time-series predicting ability, a research team from Johns Hopkins University proposed an RNN-based auxiliary framework to prevent dangerous operations during surgery [40,41]. Lin et al. developed an evaluating method for surgical operation based on a neural network [42]. Moreover, some advanced networks have great potential to enhance the performance of data-driven applications, such as the EGNN network proposed by Liu et al. [43], which can solve the problem of incomplete and noisy original data. Similarly, a novel heterogeneous network representation learning method can improve the accuracy of pattern classification [44], which can be useful for improving the safety of robot-assisted surgery. In addition, at the hardware level, some advanced sensors can endow mechatronics integration systems, especially medical robots, with more flexibility and intelligence [45]. These studies are novel and insightful, having an optimistic prospect for dental surgery.

A series of problems were solved by the above-mentioned research. However, from a broader point of view on medical robotics, as Haidegger pointed out [46], due to the complexity of the environment and task, most existing systems are implemented via master-slave operations without sufficient cognitive ability and autonomy. For instance, a neural network evaluating the surgical operation [42] still lacks decision-making intelligence, meaning that a partial autonomy method and an array of standards or protocols for automation should be fulfilled, for which Nagy et al. have performed beneficial work on this issue [47]. As mentioned before, VF is a key technology for master-slave surgery. However, current studies rarely involve VF focused on oral surgery. Automatic preoperative placement methods and VF generation for oral surgery are also limited. For example, in the study of Tang et al. [27], VF is still assigned by manual measurement. This condition lowers the fault tolerance and safety under the geometric configuration of oral surgery, since some unexpected faults may exist during manual planning. Therefore, this paper aims to propose a fast but accurate collision-detection model, and then derive an automatic method for the setup of the feeding target, as well as the previously proposed virtual fixture. The main contributions of this paper can be listed as follows:

- A new finite-parameter surrogate model (FPSM) is proposed, in which the oral cavity is replaced by a group of closed curves described in the polar coordinate system. All the parameters of closed curves can be optimized to fit the oral structure. This can significantly increase the real-time performance and reduce the computational burden for most of the planning tasks in which complex 3D oral structures should be involved.
- Based on the FPSM, an efficient collision-detection method can be determined through cylinder-to-line simplification of the instrument’s rod.
- The feeding target is automatically placed through the particle swarm optimization (PSO) algorithm, which is the second stage of optimization, forming the preoperative planning framework.
- The framework is an essential expansion of the previous work on VF. With the VF and the planning framework combined, a full strategy is established.

The rest of this paper is organized as follows. Section 2 presents the DIRS and the previously proposed VF. Section 3 introduces the FPSM, including closed curves and the first stage of optimization. Section 4 describes the collision detection, safety estimation, and the automatic placement of the feeding target based on the second stage of optimization. Simulations and results are given in Section 5. Finally, conclusions are drawn in Section 6.

## 2. System, Task, and Framework

### 2.1. System Constitution

**Figure 1:**
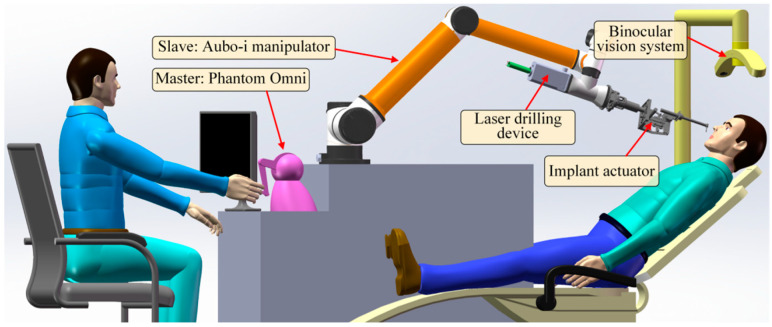
The constitution of the DIRS.

The DIRS consists of a universal manipulator (AUBO-i10), a master controller (PHANTOM Omni), an implanting actuator (driven by a FAULHABER-2036B brush-less motor and controlled by a BECKHOFF-CX2030 embedded controller), a laser drilling actuator, and a vision system (HIKVISION’s camera). The universal manipulator, which has six degrees of freedom (DOFs), carries the two actuators mounted on it. The laser drilling device is responsible for drilling a hole in the jaw bone. The implant actuator is used to place an implant into the hole. The vision system provides the position/ orientation of the oral cavity. The DIRS is shown in Figure 1.

### 2.2. Planning Task for Virtual Fixture

**Figure 2:**
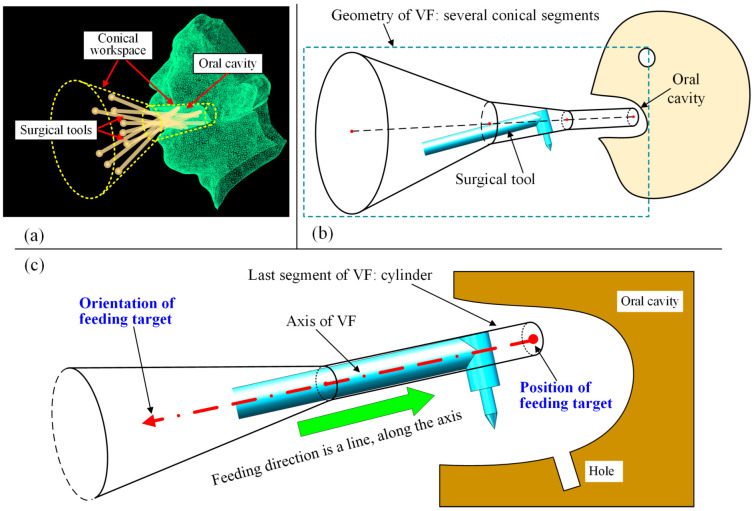
Previously proposed VF: (**a**) conical workspace for oral surgery; (**b**) geometric design of VF; (**c**) VF can be placed using a position and an orientation.

In our previously proposed work [12], the workspace is regarded as a conical space, as the instruments are inserted into the oral cavity (Figure 2a); thus, the virtual fixture consists of several conical segments (Figure 2b). Once the surgical instrument exceeds the conical space, the VF will push the instrument back to the VF, which is the safety effect of VFs. The last segment of the VF, which is mainly inside the oral cavity, is a cylinder whose radius is usually set to be the surgical tool’s radius. When the tool is inside the oral cavity, this condition makes it move along a straight line, which is the axis of the VF, and any movements beyond the VF’s axis will be hindered. Additionally, the linear movement is described relative to the oral cavity, meaning that the disturbance movement of the patient’s head, which corresponds to the motion control of the manipulator, will not be considered in this paper. In other words, all the coordinate systems and geometry objects discussed later are based on the oral cavity.

With the definition of the conical VF, the task of preoperative planning is to place the straight line, including its position and orientation. When the straight line is determined, the VF can then be formed with its axis coinciding with the line. The relationship between the VF and preoperative placement is shown in Figure 2c. It can be seen from Figure 2c that the VF can be placed using a position and an orientation, forming the feeding target to be optimized in this work.

### 2.3. Overview of Two-Stage Optimization Framework

The proposed planning framework consists of two main modules, both of which are calculated with iterative optimization. The first module is the FPSM, which represents the complex surfaces of the oral cavity using an array of fitting curves that are determined by finite parameters. By optimizing these parameters, the numerical fitting of the oral structure is performed, forming a cage-shaped surrogate model that can estimate collisions between a geometric object and the FPSM. Hence, optimizing the parameters of FPSM in the first module is the first stage of optimization.

Then, the FPSM module plays the role of collision detector, which can determine the collision status between the surgical instrument and the oral cavity both qualitatively and quantitatively, providing the objective function for the placement of the VF. By optimizing the objective function formed by the FPSM, the placement result can be obtained. Thus, optimizing the placement of the VF is the second stage of optimization.

**Figure 3:**
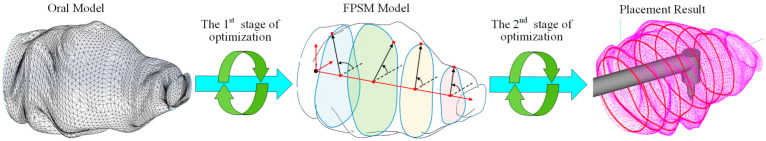
The mainline for the framework is two-stage optimization.

In a word, the first stage of optimization is the basis and prerequisite for the second stage of optimization, and the second stage of optimization provides the desired preoperative planning result directly to the users. The two-stage optimization, which is the mainline for the proposed framework, is shown in Figure 3.

## 3. Finite-Parameter Surrogate Model

**Figure 4:**
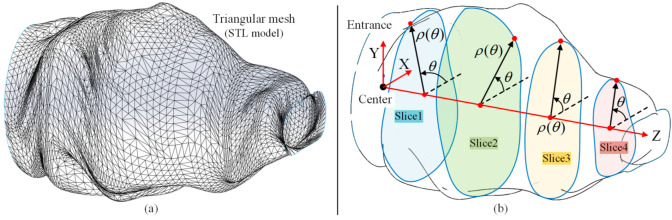
Overview of simplification of oral cavity: (**a**) triangular mesh of oral model; (**b**) finite-parameter surrogate model is a group of closed curves/slices in polar coordinates.

In this section, a FPSM is designed. Based on the triangular mesh of preoperative scanning, a set of closed curves in polar coordinates is used to simulate the triangular mesh. In other words, the oral cavity is represented by a series of parameterized slices instead of the triangular mesh, as shown in Figure 4. This section, replacing the complex triangular mesh with a simple model, is the basis of real-time collision detection, on which automatic placement of the VF is based.

### 3.1. Parameterization of Closed Curves

**Figure 5:**
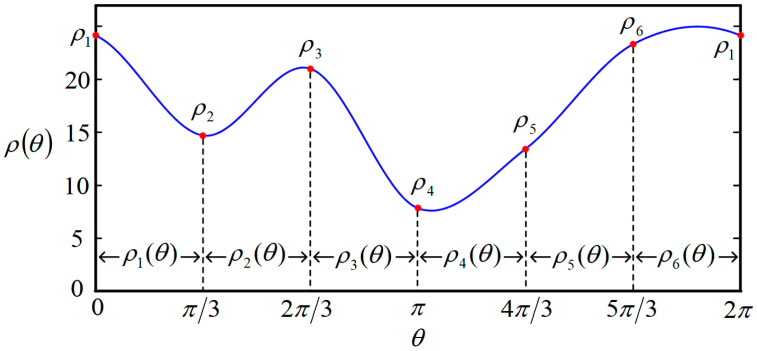
One example of closed curve function $\rho \left(\theta \right)$ when *N* = 6.

All the closed curves are described in polar coordinates, which are in the form of $\rho \left(\theta \right)$, with 0≤θ<2π always holding. The origin of the polar coordinate system is the intersection point of the Z-axis and the slice plane. The function $\rho \left(\theta \right)$ is represented by a piecewise cubic polynomial, containing *N* segments, which can better represent more complex cross-sections in the three-dimensional oral structure. Let the i-th segment of $\rho \left(\theta \right)$ be $\rho_{i}\left(\theta \right)$, seen as follows.

(1) $$\rho_{i}\left(\theta \right)=a_{i}+b_{i}\theta +c_{i}\theta^{2}+d_{i}\theta^{3}\;\frac{2\pi \left(i-1\right)}{N}\leq \theta <\frac{2\pi \;i}{N}$$

where $a_{i}$, $b_{i}$, $c_{i}$, and $d_{i}$ are the polynomial coefficients of $\rho_{i}\left(\theta \right)$. *N* is the number of segments of $\rho \left(\theta \right)$. The larger *N* is, the more undetermined parameters there are, but the fitting capacity is higher. Contrariwise, as *N* becomes less, fewer parameters have to be solved. However, the fitting accuracy will be lower. When *N* = 6, one example of the closed curve function $\rho \left(\theta \right)$, described in polar coordinates, can be seen in Figure 5.

Since $\rho \left(\theta \right)$ is formed of several segments, some constraints must be satisfied at the connecting points between two segments. As a continuous and smooth function, the function values and derivative values are continuous at the connection points. Additionally, to avoid the oscillation of the function, the derivative values at the connection points are designed to be equal to the average change rate among two adjacent segments. These constraints can be written as

(2) $$\left\{\begin{array}{l} \rho_{i}\left(2\pi \;i/N\right)=\rho_{i+1}\left(2\pi \;i/N\right)=\rho_{i+1}\\ \rho_{i}^{\prime }\left(2\pi \;i/N\right)=\rho_{i+1}^{\prime }\left(2\pi \;i/N\right)={\overset{-}{v}}_{i,i+1}\\ {\overset{-}{v}}_{i,i+1}=\frac{N\left(\rho_{i+2}-\rho_{i}\right)}{4\pi } \end{array}\right.$$

where i is the index of the i-th segment with 1≤i≤N. ${\bar{v}}_{i,i+1}$ denotes the average derivative value of segments i and i+1. What has to be explained here is that the index is cyclical, that is to say, the circumstance where i=N+1 is equal to i=1, and the circumstance where i=N+2 can be replaced by i=2. This definition provides how to deal with the out-of-range index for Equation (2).

**Figure 6:**
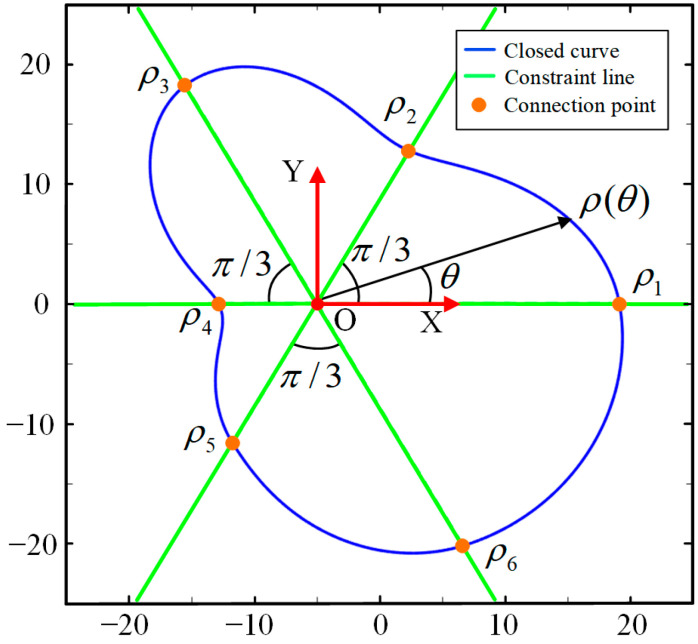
Illustration of the closed curve when *N* = 6.

It can be seen from Equation (1) that there are 4*N* undetermined parameters to obtain a closed curve. Also, 4N constraints exist in Equation (2), which means the closed curve $\rho \left(\theta \right)$ can be obtained by solving the linear equation, as shown in Equation (3).

(3) $$\left\{\begin{array}{l} M\cdot a=b\\ M=\left[M_{\rho };M_{v}\right]\\ a={\left[a_{1},b_{1},c_{1},d_{1},a_{2},b_{2},c_{2},d_{2},\cdots c_{N},d_{N}\right]}^{\;T}\\ b={\left[b_{\rho },b_{v}\right]}^{\;T} \end{array}\right.$$

where a is the coefficient vector that includes all the required polynomial coefficients to determine the whole closed curve $\rho \left(\theta \right)$. M is the constraint matrix assembled using the function value constraint matrix $M_{\rho }$ and the derivative value constraint matrix $M_{v\;}$, as shown in Equation (4). b is the constraint vector accompanied by M, which is assembled using the function value constraint vector $b_{\rho }$ and the derivative value constraint vector $b_{v\;}$, as shown in Equation (5). All polynomial coefficients can be obtained by solving the equation M·a=b.

(4) $$\begin{array}{l} M_{\rho }=\left[\begin{array}{ccc} P_{0} & & O\\ & \ddots & \\ O & & P_{N-1} \end{array}\right]\;M_{v}=\left[\begin{array}{ccc} Q_{0} & & O\\ & \ddots & \\ O & & Q_{N-1} \end{array}\right]\\ b_{\rho }=\left[\rho_{1}\;\rho_{2}\;\rho_{2}\;\rho_{3}\;\rho_{3}\cdots \rho_{N}\;\rho_{N}\;\rho_{1}\right]\\ b_{v}=\left[\begin{array}{ccccccc} {\overset{-}{v}}_{N,1} & {\overset{-}{v}}_{1,2} & {\overset{-}{v}}_{1,2} & \cdots & {\overset{-}{v}}_{N-1,N} & {\overset{-}{v}}_{N-1,N} & {\overset{-}{v}}_{N,1} \end{array}\right] \end{array}$$

where $\rho_{1},\;\rho_{2},\;\rho_{3},\;\cdots \rho_{6}$ can be found in Figure 5 and Figure 6, which are the ρ values of the connecting points, and can be adjusted to change the function $\rho \left(\theta \right)$. ${\bar{v}}_{i,i+1}$ is the same symbol explained in Equation (2). $P_{j}$ and $Q_{j}$ are constant matrices to assemble the constraint matrix M, shown as follows.

(5) $$\begin{array}{l} P_{i}=\left[\begin{array}{cccc} 1 & \frac{2\pi \;i}{N} & {\left(\frac{2\pi \;i}{N}\right)}^{2} & {\left(\frac{2\pi \;i}{N}\right)}^{3}\\ 1 & \frac{2\pi \left(i+1\right)}{N} & {\left(\frac{2\pi \left(i+1\right)}{N}\right)}^{2} & {\left(\frac{2\pi \left(i+1\right)}{N}\right)}^{3} \end{array}\right]\\ Q_{i}=\left[\begin{array}{cccc} 0 & 1 & \frac{4\pi \;i}{N} & 3\;{\left(\frac{2\pi \;i}{N}\right)}^{2}\\ 0 & 1 & \frac{4\pi \left(i+1\right)}{N} & 3\;{\left(\frac{2\pi \left(i+1\right)}{N}\right)}^{2} \end{array}\right] \end{array}$$

At this point, the parameterization process for the closed curve is conducted, that is, $\rho_{1},\;\rho_{2},\;\rho_{3},\;\cdots \rho_{N}$ (or *N* control points) are the parameters that can fully determine a closed curve/slice, and the closed curve can be intuitively deformed to suit the 3D structure/boundary according to these ρ values. As shown in Figure 6, when N=6, the six connecting points (or control points) are located on the constraint lines. By adjusting the polar radius $\rho_{1},\;\rho_{2},\;\rho_{3},\;\cdots \rho_{6}$, the shape can be randomly changed. Therefore, to find the best closed curve, the problem is converted to finding the best combination of the six polar radii $\rho_{1},\;\rho_{2},\;\rho_{3},\;\cdots \rho_{6}$.

### 3.2. Parameter Optimization of Closed Curves

As introduced in Section 3.1, when the closed curves are parameterized, the shape of the closed curve will be uniquely determined by the N control points. However, the FPSM cannot be adopted until it is optimized to make the closed curve fitted to the oral structure. On the one hand, if the volume of the FPSM is obviously smaller than the oral structure, many feasible spaces for VF placement will no longer accessible for planning. On the other hand, if the FPSM is apparently larger than the oral cavity, the planning result may well penetrate the surface of the oral cavity, causing risks of damaging the tissue. Following these reasons, there are two principles of objective function for optimization (stage 1):

1. The area of the closed curve should be as large as possible to better-simulate the boundary of the oral structure, and the maximum possible area is when the closed curve exactly coincides with the boundary. This point aims to provide accessible spaces for preoperative planning as much as possible.
2. The closed curve should not collide with the triangular mesh, or should only allow very slight collisions to be accepted. This point aims to prevent collisions and ensure safety.

In the framework, optimization for closed curves of the FPSM is the first stage of optimization, and its iterative pattern is presented as follows: let the area of a closed curve be S; after all the polynomial coefficients $a_{i}$, $b_{i}$, $c_{i}$, and $d_{i}$ are obtained by using Equation (3), the area S can be obtained as follows

(6) $$\begin{array}{l} S=\frac{1}{2}\int_{0}^{2\pi }\rho {\left(\theta \right)}^{2}\;d\theta \\ \;=\frac{1}{2}\overset{N}{\underset{i=1}{\sum }}\left(a_{i}^{2}\theta +a_{i}b_{i}\theta^{2}+\frac{2a_{i}c_{i}+b_{i}^{2}}{3}\theta^{3}+\frac{a_{i}d_{i}+b_{i}c_{i}}{2}\theta^{4}\right.\\ \;{\left.\left.+\frac{2b_{i}d_{i}+c_{i}^{2}}{5}\theta^{5}+\frac{c_{i}d_{i}}{3}\theta^{6}+\frac{d_{i}^{2}}{7}\theta^{7}\right)\right|}_{\frac{2\pi i}{N}}^{\frac{2\pi \left(i-1\right)}{N}} \end{array}$$

Except for the area S, which is the one important part of the optimization objective, another requirement is to avoid interference or only accept very slight interference. Following this requirement, the penalty function method is adopted, serving as another part of the optimization objective. To evaluate the extent of interference between the closed curve and the triangular mesh, Z sampling points are evenly distributed on $\rho \left(\theta \right)$. By counting the number of sampling points penetrating the boundary $Z_{\mathrm{pene}}$, which can obtain the rate of interference $f_{\mathrm{pene}}=Z_{\mathrm{pene}}/Z$, the extent of the collision can be estimated. For example, if one sets Z to be 360, then the angles of the sampling points are 0°, 1°,…,359°; when 36 out of these 360 sampling points penetrate the boundary, $f_{\mathrm{pene}}=0.1$. Then, let $\lambda_{\mathrm{pene}}\;\left(\lambda_{\mathrm{pene}}>0\right)$ be the penalty coefficient. The optimization objective function $F_{\mathrm{aim}}$ can be written as

(7) $$F_{\mathrm{aim}}=-S+\lambda_{\mathrm{pene}}f_{\mathrm{pene}}^{2}$$

where $\lambda_{\mathrm{pene}}f_{\mathrm{pene}}^{2}$ is the penalty term to reduce the collision.

In Equation (7), when S is getting larger or $f_{\mathrm{pene}}$ is getting smaller, the performance is better, or the performance will get worse otherwise. Generally, to better-prevent interference, the penalty coefficient $\lambda_{\mathrm{pene}}$ should be relatively bigger, such as $\lambda_{\mathrm{pene}}=100,000$. In that case, even a very slight interference can trigger a significant increase in the penalty term $\lambda_{\mathrm{pene}}f_{\mathrm{pene}}^{2}$, which can make the performance $F_{\mathrm{aim}}$ become much worse. Thus, the closed curve will not penetrate the boundary, or only a very small contact with the boundary will occur. Then, the objective function can be optimized by using the gradient descent method, as shown in Equation (8).

(8) $$\rho_{k+1}=\rho_{k}-\alpha \frac{\frac{\partial F_{\mathrm{aim}}}{\partial \rho_{k}}}{\left||\frac{\partial F_{\mathrm{aim}}}{\partial \rho_{k}}|\right|}$$

where $\rho_{k}$ is the ρ value in the k-th round of iteration. α is the size of the iteration steps, such as α=0.1. For the partial derivative term, it can be approximately calculated using numerical differentiation:

(9) $$\frac{\partial F_{\mathrm{aim}}}{\partial \rho_{i}}\approx \frac{F_{\mathrm{aim}}\left(\rho_{i}+\Delta \rho \right)-F_{\mathrm{aim}}\left(\rho_{i}-\Delta \rho \right)}{2\Delta \rho }$$

**Figure 7:**
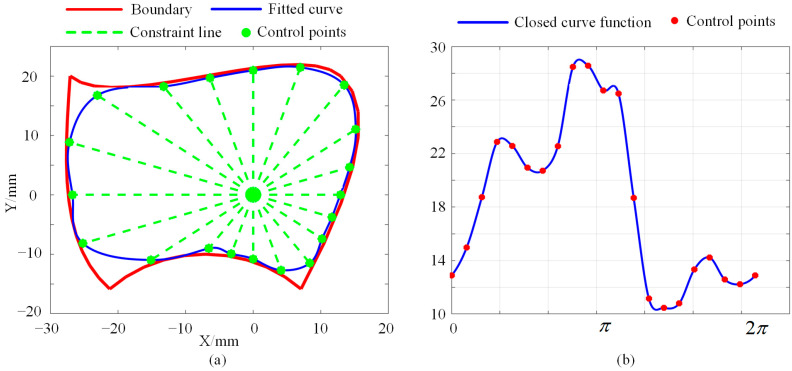
Example of parameter optimization for boundary fitting: (**a**) Optimized closed curve. (**b**) The $\rho \left(\theta \right)$ function for the closed curve.

With the optimization process, one slice can be determined. Another example is shown in Figure 7a, where a predefined boundary is drawn in red, and the optimized curve for fitting the boundary is drawn in blue. It can be seen from Figure 7a that there are 20 control points being used, meaning that 20 parameters are involved in this slice. The $\rho \left(\theta \right)$ function for the closed curve is shown in Figure 7b. This case illustrates that the complex boundary can be approximately fitted by another simple curve, which is adequate for collision detection. This will be discussed in Section 5.

### 3.3. Establishment of FPSM

In Section 3.1 and Section 3.2, a single slice can be established. When an array of slices are arranged evenly through the oral cavity, the triangular mesh can be replaced by the group of slices, as shown in Figure 4. Letting the number of slices be M, there are M polar coordinate systems whose origins coincide with the Z-axis in Figure 4.

**Figure 8:**
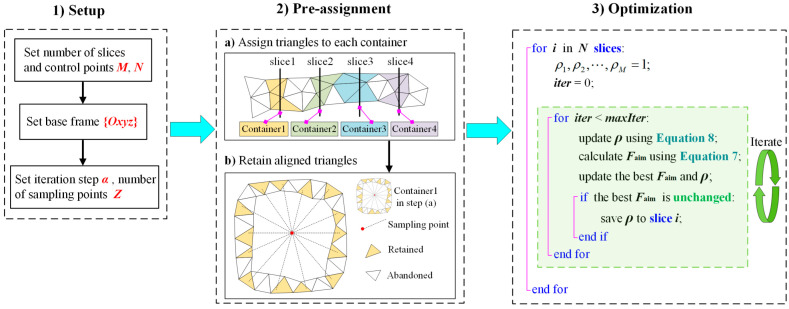
Procedures for establishing the FPSM.

To accelerate the establishment of the FPSM, a pre-assignment step is important. For a slice, it corresponds to a container, and all triangles intersecting with the slice will be put into its container. Then, for triangles in the container, only triangles aligned with Z sampling points (see the definition of $f_{\mathrm{pene}}$ in Equation (7)) will be retained, and the others will be abandoned. Therefore, during the optimization, only triangles in the containers will be involved, avoiding the calculation of the entire triangular mesh, which can accelerate the optimization to a great extent. The steps to establish the FPSM are listed as follows, and are also seen in Figure 8.

## 4. Automatic Placement of Feeding Target

In Section 3, the FPSM was obtained by the first stage of optimization. Based on the FPSM, an optimization-based preoperative placement method is presented, which is the second optimization process for the preoperative framework. In this section, an objective function is designed via collision detection and safety evaluation using the FPSM. Then, undetermined parameters of the feeding target are extracted and optimized by the PSO algorithm, obtaining the best position/orientation to place the virtual fixture.

### 4.1. Collision Detection and Safety Estimation

**Figure 9:**
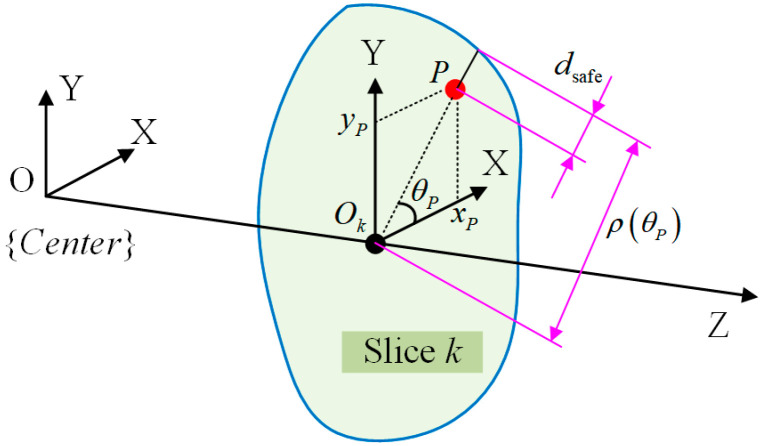
Collision detection for a single point.

For collision detection, the relationship between the FPSM and spatial elements (e.g., point, line, cylinder) is the core. As shown in Figure 9, for a point *P* coinciding with a slice whose closed curve is $\rho \left(\theta \right)$, the safe distance is

(10) $$d_{\mathrm{safe}}=\rho \left(\theta_{P}\right)-\sqrt{x_{P}^{2}+y_{P}^{2}}$$

where $d_{\mathrm{safe}}^{\left(k\right)}$ is the safe distance, which is the remaining distance from penetrating the FPSM. $x_{P}$, $y_{P}$, and $\theta_{P}$ are shown in Figure 9. If $d_{\mathrm{safe}}>0$, point *P* is regarded to be inside the boundary; otherwise, a collision is detected. If point *P* is located between two adjacent slices $S_{1}$ and $S_{2}$, the safe distance can be obtained via linear interpolation.

(11) $$d_{\mathrm{safe}}=d_{\mathrm{safe}}^{\left(S1\right)}+\frac{\left(d_{\mathrm{safe}}^{\left(S2\right)}-d_{\mathrm{safe}}^{\left(S1\right)}\right)\;d_{P,S1}}{d_{S1,S2}}$$

where $d_{\mathrm{safe}}^{\left(S1\right)}$ and $d_{\mathrm{safe}}^{\left(S2\right)}$ are safe distances of *P*’s projection points on $S_{1}$ and $S_{2}$, $d_{S1,S2}$ is the distance between $S_{1}$ and $S_{2}$, and $d_{P,S1}$ is the distance between P and $S_{1}$.

**Figure 10:**
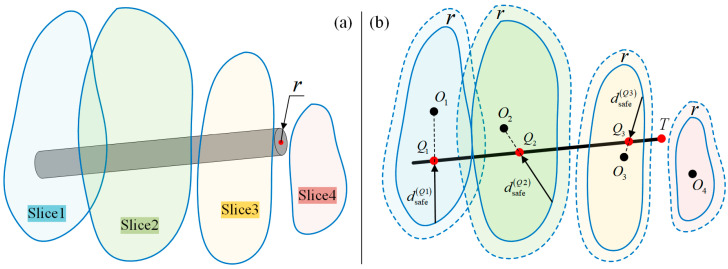
Collision detection for a line/rod: (**a**) Rod and FPSM. (**b**) Shrink the FPSM for collision detection, which is collision detection between a line and the FPSM.

For a line segment *L*, assume *m* slices are intersected by line *L*. The intersection points are $Q_{1},Q_{2},\cdots ,Q_{m}$, as shown in Figure 10b. Qualitatively, when $d_{\mathrm{safe}}^{\left(Q1\right)}$, $d_{\mathrm{safe}}^{\left(Q2\right)}$,…, $d_{\mathrm{safe}}^{\left(Qm\right)}$ are all larger than zero, line *L* is regarded as collision-free. If one or more of the $d_{\mathrm{safe}}$ values is less than or equal to zero, a collision is detected.

Then, for a cylinder shown in Figure 10a, collision detection is similar. Letting the radius be r, by simplifying the rod into a line while shrinking the closed curve inward by r (that is, shrink the finite-parameter surrogate model by r), the collision detection of a rod is just the same as a line, as shown in Figure 10b.

(12) $$\rho^{*}\left(\theta \right)=\rho \left(\theta \right)-r$$

where $\rho^{*}\left(\theta \right)$ is the closed curve when the FPSM is shrunk by r. The polar coordinate of the FPSM is quite convenient. Collision detection for a rod is illustrated in Figure 10.

The above steps can qualitatively check whether a collision occurs. However, to optimize the placement of the VF, a method that can quantificationally evaluate the safety performance is required, for which a safety indicator should be designed. Here, the safety indicator of a rod can be written as:

(13) $$f_{\mathrm{safe}}=\left\{\begin{array}{ll} \frac{\lambda_{\mathrm{avg}}}{m+1}\left(d_{\mathrm{safe}}^{\left(T\right)}+\sum_{i=1}^{m}d_{\mathrm{safe}}^{\left(Qi\right)}\right)+\lambda_{\min}d_{\mathrm{safe}}^{\left(\min\right)} & \left(d_{\mathrm{safe}}^{\left(\min\right)}>0\right)\\ \lambda_{\min}d_{\mathrm{safe}}^{\left(\min\right)} & \left(d_{\mathrm{safe}}^{\left(\min\right)}\leq 0\right) \end{array}\right.$$

where $f_{\mathrm{safe}}$ is the safe indicator for a line/rod and $d_{\mathrm{safe}}^{\left(\min\right)}=\min\;\{d_{\mathrm{safe}}^{\left(Q1\right)},d_{\mathrm{safe}}^{\left(Q2\right)},\cdots ,d_{\mathrm{safe}}^{\left(Qm\right)},d_{\mathrm{safe}}^{\left(T\right)}\}$ is the minimum safe distance among m+1 safe distances. m is the number of intersection points between the line and the slices, along with the endpoint *T*, forming the m+1 sampling points to decide whether a collision occurs. The first term using $\lambda_{\mathrm{avg}}$ is the average value term and the second term using $\lambda_{\min}$ is the extreme value term. $\lambda_{\mathrm{avg}}$ and $\lambda_{\min}$ are the weighting coefficients of the average and minimum safe distance.

In Equation (13), when a collision is detected, it can be asserted that $d_{\mathrm{safe}}^{\left(\min\right)}\leq 0$ always holds true. In this case, the average value term loses its meaning, and only the extreme value term will remain in Equation (13), which can not only qualitatively determine whether a collision occurs, but can also quantificationally assess the extent of the collision. Contrariwise, when no collision occurs, $d_{\mathrm{safe}}^{\left(\min\right)}\geq 0$ always holds true, and the indicator $f_{\mathrm{safe}}$ must be larger than 0. The larger $f_{\mathrm{safe}}$ is, the safer the VF can be. Briefly, if $f_{\mathrm{safe}}$ is larger than zero, the situation is collision-free, or a collision is detected. For $f_{\mathrm{safe}}$, the larger, the better, the smaller, the worse.

### 4.2. Establishment of Objective Function

To determine which position/orientation the VF should be placed in, the safety should be quantificationally evaluated, establishing the objective function by using the collision detection model discussed in Section 4.1.

**Figure 11:**
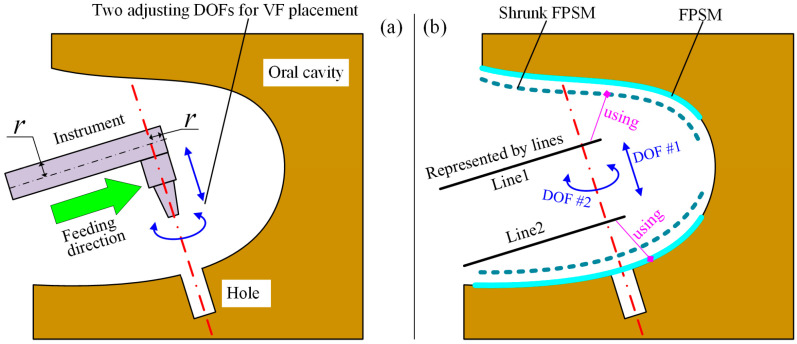
Collision detection for feeding process: (**a**) Two adjustable DOFs. (**b**) Geometry model to build objective function.

As illustrated in Figure 11a, the feeding direction is along the rod’s axis. After the tool is aligned with the hole, only two DOFs can adjust the placement of the VF: linear movement along the hole’s axis, and rotation around the hole’s axis. By adjusting the two DOFs, a relatively safer preoperative setting of the VF can be found.

In Figure 11b, the rod is simplified to line1, with the FPSM and its shrunk model shown. In addition, line2 is defined for obstacle avoidance. This is because the space swept by the feeding motion along line1 may collide with the tissue. In other words, the geometry model should represent the whole occupied space rather than one instant. Following the geometry model, the objective function for VF placement can be built as:

(14) $$F_{\mathrm{safe}}=-\frac{\left(m_{1}+1\right)sgn\left(f_{\mathrm{safe}}^{\left(L1\right)}\right)\sqrt{\left|f_{\mathrm{safe}}^{\left(L1\right)}\right|}+\left(m_{2}+1\right)sgn\left(f_{\mathrm{safe}}^{\left(L2\right)}\right)\sqrt{\left|f_{\mathrm{safe}}^{\left(L2\right)}\right|}}{m_{1}+m_{2}+2}$$

where $F_{\mathrm{safe}}$ is the objective function for placement. $f_{\mathrm{safe}}^{\left(L1\right)}$ and $f_{\mathrm{safe}}^{\left(L2\right)}$ are, respectively, the $f_{\mathrm{safe}}$ value for line1 and line2 calculated using Equation (13). $m_{1}$ are $m_{2}$ are the number of intersection points of line1 and line2. The two fractions, decided by $m_{1}$ and $m_{2}$, are the weights of $f_{\mathrm{safe}}^{\left(L1\right)}$ and $f_{\mathrm{safe}}^{\left(L2\right)}$.

During the second stage of optimization, whose target is Equation (14), when $f_{\mathrm{safe}}^{\left(L1\right)}$ and $f_{\mathrm{safe}}^{\left(L2\right)}$ are all larger than 0, a collision-free solution is found, or the current placement cannot be accepted. The smaller $F_{\mathrm{safe}}$ is, the better the placement of the VF. After the iterative algorithm (the second stage of optimization) returns its result, if $f_{\mathrm{safe}}^{\left(L1\right)}\leq \;$0 or $f_{\mathrm{safe}}^{\left(L2\right)}\leq \;$0, the placement result is invalid and the optimization has failed.

Further, $f_{\mathrm{safe}}^{\left(L2\right)}$ should be calculated by using the none-shrunk FPSM to avoid unnecessary adjustment since line2 is not a rod, as is indicated in Figure 11b.

### 4.3. Optimization of Preoperative Placement

**Figure 12:**
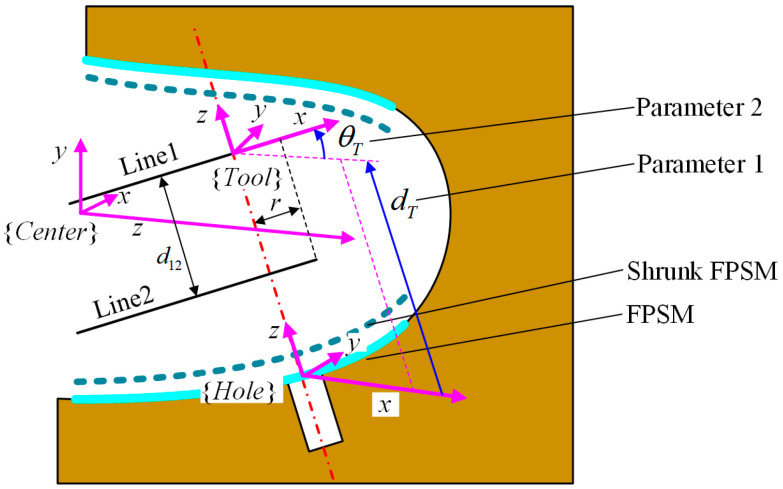
Frame definition and parameters for optimization.

To obtain a relatively satisfied placement of the implant tool and VF, the second stage of optimization is responsible, where $d_{T}$ and $\theta_{T}$ should be optimized, which are translational and rotational parameters along the z-axis of $\left\{Hole\right\}$, as shown in Figure 12.

For this stage of optimization, the objective function $F_{\mathrm{safe}}$ is critical. However, $F_{\mathrm{safe}}$ cannot be obtained until it is connected to $d_{T}$ and $\theta_{T}$. In Figure 12, the frame $\left\{Tool\right\}$ is defined with its x-axis along line1 and its z-axis along the rotating axis. The definition of frames and undetermined parameters for optimization are shown in Figure 12.

In terms of frame $\left\{Hole\right\}$, its origin is expressed in $\left\{Center\right\}$, which can be written as $t_{ch}$. The z-axis of $\left\{Hole\right\}$ coincides with the hole’s axis, whose unit vector is $D_{hz}={\left(D_{hz}^{\left(x\right)},\;\;D_{hz}^{\left(y\right)},\;D_{hz}^{\left(z\right)}\right)}^{T}$, described in $\left\{Center\right\}$, and its x-axis is parallel with the YZ plane in frame $\left\{Center\right\}$. Let the x-axis of $\left\{Hole\right\}$ be $D_{hx}={\left(D_{hx}^{\left(x\right)},\;\;D_{hx}^{\left(y\right)},\;D_{hx}^{\left(z\right)}\right)}^{T}$, expressed in $\left\{Center\right\}$, which is a unit vector. From the definition, $D_{hx}^{\left(x\right)}=0$ holds. Because $D_{hx}$ is perpendicular to $D_{hz}$, the relationship between $\;D_{hx}^{\left(y\right)}$ and $D_{hx}^{\left(z\right)}$ is

(15) $$\left\{\begin{array}{l} D_{hx}^{\left(y\right)}D_{hz}^{\left(y\right)}+D_{hx}^{\left(z\right)}D_{hz}^{\left(z\right)}=0\\ D_{hx}^{{\left(y\right)}^{2}}+D_{hx}^{{\left(z\right)}^{2}}=1 \end{array}\right.$$

By solving Equation (15), $D_{hx}^{\left(y\right)}$ and $D_{hx}^{\left(z\right)}$ can be obtained as follows.

(16) $$\left\{\begin{array}{l} D_{hx}^{\left(z\right)}=\frac{\left|D_{hz}^{\left(y\right)}\right|}{\sqrt{D_{hz}^{{\left(y\right)}^{2}}+D_{hz}^{{\left(z\right)}^{2}}}}\\ D_{hx}^{\left(z\right)}=-\frac{D_{hz}^{\left(z\right)}}{D_{hz}^{\left(y\right)}}\frac{\left|D_{hz}^{\left(y\right)}\right|}{\sqrt{D_{hz}^{{\left(y\right)}^{2}}+D_{hz}^{{\left(z\right)}^{2}}}} \end{array}\right.$$

With $D_{hx}$ and $D_{hz}$ already known, the rotation matrix from $\left\{Center\right\}$ to $\left\{Hole\right\}$, which is $R_{ch}$, can be seen as follows:

(17) $$R_{ch}=\left(\begin{array}{ccc} D_{hx} & D_{hz}\times D_{hx} & D_{hz} \end{array}\right)$$

Then, the transformation matrix $T_{ct}$ from $\left\{Center\right\}$ to $\left\{Tool\right\}$ can be written as:

(18) $$T_{ct}=T_{ch}\cdot T_{ht}=\left(\begin{array}{cc} R_{ch} & t_{ch}\\ 0 & 1 \end{array}\right)\cdot \left(\begin{array}{cc} R_{ht} & t_{ht}\\ 0 & 1 \end{array}\right)=\left(\begin{array}{cc} R_{ch}\cdot R_{ht} & R_{ch}\cdot t_{ht}+t_{ch}\\ 0 & 1 \end{array}\right)$$

where $T_{ch}$ is the transformation matrix from $\left\{Center\right\}$ to $\left\{Hole\right\}$ assembled by $R_{ch}$ and $t_{ch}$. $t_{ch}$ is defined based on the 3D model of the oral cavity. $T_{ht}$ is the transformation matrix from $\left\{Hole\right\}$ to $\left\{Tool\right\}$ assembled by $R_{ht}$ and $t_{ht}$. $R_{ht}$ is the rotation matrix from $\left\{Hole\right\}$ to $\left\{Tool\right\}$, which can represent the rotation around the z-axis by $\theta_{T}$. $t_{ht}$ is the translational vector ${\left[0,\;0,\;d_{T}\right]}^{T}$ decided by $d_{T}$, representing the movement from $\left\{Hole\right\}$ to $\left\{Tool\right\}$. Hence, the endpoint and the direction of *line*1 and *line*2 can be obtained as:

(19) $$\left\{\begin{array}{l} D_{\mathrm{line}}^{\left(1\right)}=R_{ch}\cdot {\left[cos\theta_{T},\;sin\theta_{T},\;0\right]}^{T}\\ D_{\mathrm{line}}^{\left(2\right)}=R_{ch}\cdot {\left[cos\theta_{T},\;sin\theta_{T},\;0\right]}^{T}\\ {\left[P_{\mathrm{line}}^{\left(1\right)},\;1\right]}^{T}=T_{ct}\cdot {\left[r,\;0,\;0,\;1\right]}^{T}\\ {\left[P_{\mathrm{line}}^{\left(2\right)},\;1\right]}^{T}=T_{ct}\cdot {\left[r,\;0,-d_{12},\;1\right]}^{T} \end{array}\right.$$

where $\;D_{\mathrm{line}}^{\left(1\right)}$ and $\;D_{\mathrm{line}}^{\left(2\right)}$ are the unit direction vector of line1 and line2, and also the first column of the rotation matrix $R_{ch}R_{ht}$. $\;P_{\mathrm{line}}^{\left(1\right)}$ and $\;P_{\mathrm{line}}^{\left(2\right)}$ are the positions of the endpoint of line1 and line2. r is the radius of the tool. $d_{12}$ is the length of the implant. With Equation (19), all the intersection points can be obtained, which can calculate the objective function in Equation (14). At this point, objective $F_{\mathrm{safe}}$ is connected to $d_{T}$ and $\theta_{T}$, given that line1 and line2 can be expressed by them.

**Figure 13:**
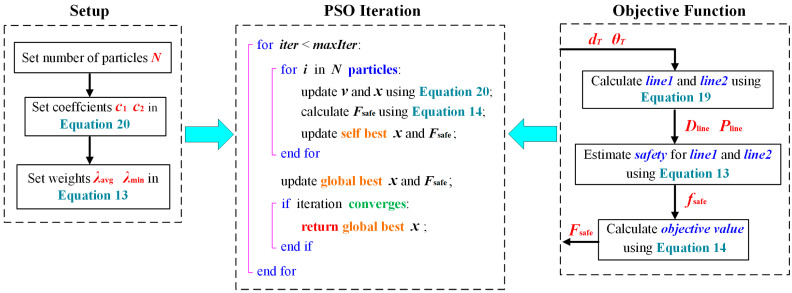
Process of preoperative placement optimization.

To find a good combination of $d_{T}$ and $\theta_{T}$, an effective optimization algorithm must be used. Because the objective $F_{\mathrm{safe}}$ is discontinuous, whose performance is sensitive to the initial value for iteration, gradient-based algorithms are not palatable. Thus, as one of the most powerful bionic algorithms, the PSO algorithm was selected to find a satisfied preoperative placement. The iteration for PSO can be written as:

(20) $$\left\{\begin{array}{l} v_{k+1}^{\left(i\right)}=c_{0}v_{k}^{\left(i\right)}+r_{1}c_{1}\left(x_{\mathrm{best}}^{\left(i\right)}-x_{k}^{\left(i\right)}\right)+r_{2}c_{2}\left(x_{\mathrm{best}}^{\left(glb\right)}-x_{k}^{\left(i\right)}\right)\\ x_{k+1}^{\left(i\right)}=x_{k}^{\left(i\right)}+v_{k+1}^{\left(i\right)} \end{array}\right.$$

where $x_{k}^{\left(i\right)}$ and $v_{k}^{\left(i\right)}$ are, respectively, the position and velocity of particle i in the k-th iteration, with $x={\left[d_{T}\;,\;\theta_{T}\right]}^{T}$. $c_{0}$, $c_{1}$, and $c_{2}$ are, respectively, the inertia, self-cognition, and group-cognition coefficients. $r_{1}$ and $r_{2}$ are random values ranging from 0 to 1. $x_{\mathrm{best}}^{\left(i\right)}$ and $x_{\mathrm{best}}^{\left(glb\right)}$ are, respectively, the best position of particle i and the best position among the entire particle swarm. The optimization process is shown in Figure 13.

## 5. Tests, Results, and Discussion

**Figure 14:**
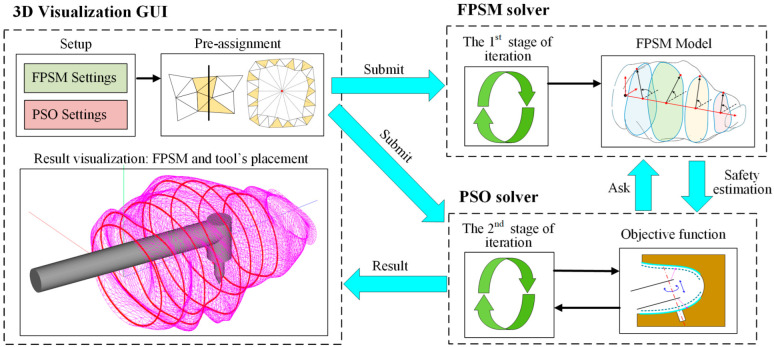
The full framework of proposed method, whose core is two-stage optimization.

Based on Section 3 and Section 4, the FPSM solver, the PSO solver, and a 3D visualization software (GUI) were developed in C++ language, with another FPSM program written in MATLAB language for detailed testing. All the programs were run on a PC with Intel(R) Core(TM) i5-10200H at 2.4 GHz and 16 GB RAM in a 64-bit win10 system. In the implementation, the settings of FPSM and PSO were input through GUI and submitted to the FPSM solver and PSO solver. Also, the pre-assignment was performed through GUI and submitted to the FPSM solver. After the FPSM was solved in the first stage of optimization, it played the role of collision detection and safety estimation. Then, based on the FPSM, the implanting tool’s placement could be solved by the PSO solver. Finally, the result was displayed in the GUI. The full framework is shown in Figure 14.

### 5.1. Test of FPSM Establishment

**Figure 15:**
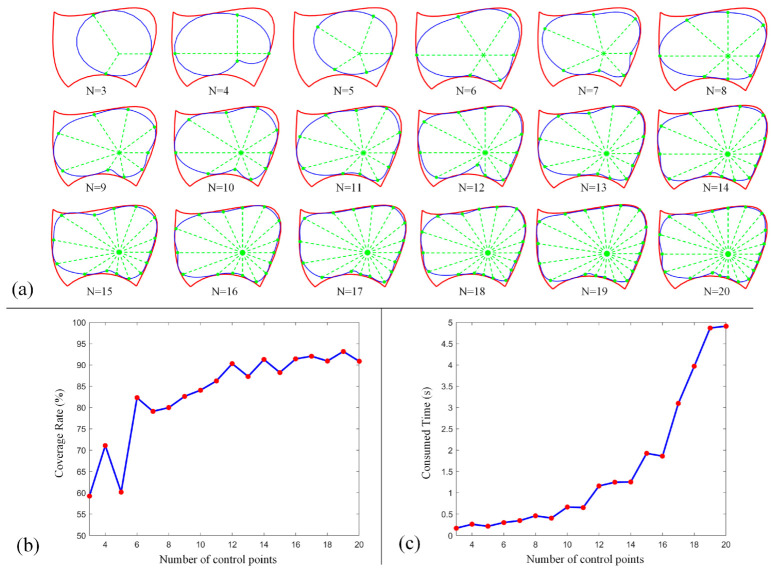
Performance of FPSM when different control points are involved: (**a**) Shape of closed curve. (**b**) Coverage rate of a close curve. (**c**) Time consumption to optimize a closed curve.

In this section, the FPSM will be tested using different parameter settings, evaluating its performance, such as for coverage rate and time consumption. Firstly, for a closed curve (slice), the number of control points N (Equation (1)) is a critical parameter. When the number is larger, the coverage rate inclines higher, and the fitting is better. However, the time consumption tends to be larger when more control points are involved. By adopting the boundary in Figure 7a, we ran the MATLAB version of the program on MATLAB R2017b environment; the results of the coverage rate and time consumption are listed in Table 1. Additionally, the performance data are drawn in Figure 15, including shapes (Figure 15a), coverage rate (Figure 15b), and consumed time (Figure 15c). In this test, other settings were $\lambda_{p}=20,000\;$(Equation (7)) and α=1.0 (Equation (8)).

From these data, the main tendency is for the coverage rate to increase when more control points are involved, although not all the data support this tendency. However, when the number is larger than 15, such an increase will slow down. Also, as more control points are involved, the consumed time increases significantly, especially when the number is larger than 15. To balance the coverage rate and time consumption, the number of control points can be set to 15, which can cover most of the boundary with relatively less time consumption.

**Figure 16:**
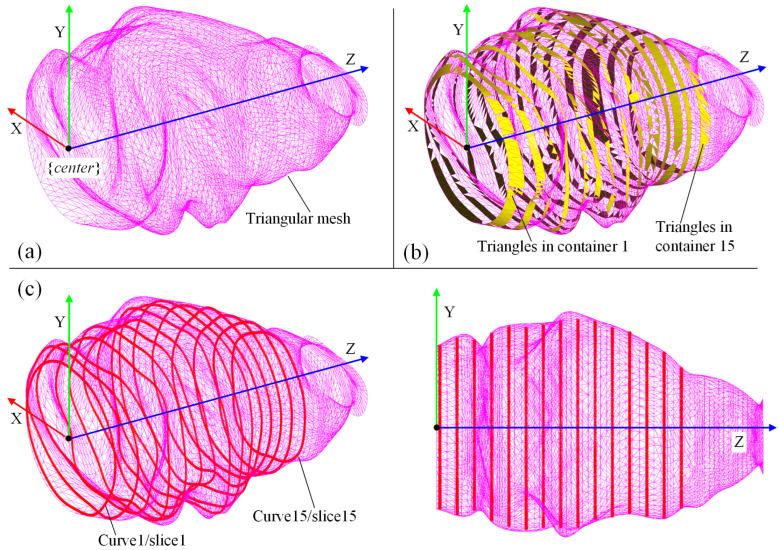
Result of the C++ version of FPSM establishment: (**a**) Model of oral cavity. (**b**) Result of pre-assignment for triangular mesh. (**c**) The FPSM is optimized and displayed.

Then, we used the C++ version of the FPSM solver, which loads a 3D model of the oral cavity in oral format, and ran the GUI and solver with the following settings: 15 slices, 15 control points for each slice, 150 sampling points for evaluating the collision of each slice, the penalty coefficient $\lambda_{p}$ was 30,000, the iteration step length α was 0.1, and the maximum number of iterations was 1000. It took the solver 33.613 s to optimize the entire model, which consisted of 15 slices. The results are shown in Figure 16, including the triangular mesh of the oral cavity (Figure 16a), the highlighting of triangles selected by pre-assignment (Figure 16b), and the optimized FPSM (Figure 16c). In Figure 16, the closed curves can well-fit the triangular mesh, meaning that these closed curves can represent the complex triangular mesh, indicating that the FPSM is an effective substitute for the mesh.

### 5.2. Test of Optimization for Instrument Placement

**Figure 17:**
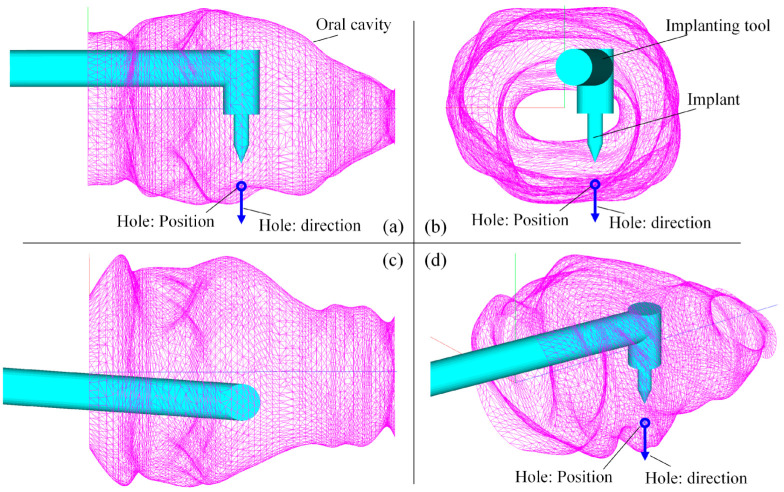
Result of preoperative placement obtained from PSO solver: (**a**) YZ view. (**b**) XY view. (**c**) XZ view. (**d**) Isometric view.

As mentioned before, the FPSM serves as a fast model for collision detection and safety estimation. With the FPSM, the position/orientation of the surgical tool’s target can be determined via the second stage of optimization. The settings for the implanting task were as follows: the radius of the rod was 6 mm, $d_{12}$ was 31 mm, and the position and direction of the implanting hole were $t_{ch}={\left[-10,-25,\;50\right]}^{T}$ and $D_{hz}={\left[0,1,\;0\right]}^{T}$, expressed in $\left\{hole\right\}$. For the PSO solver, the settings were 30 particles, $c_{1}=0.3$, $c_{2}=0.4$, $\lambda_{\mathrm{avg}}=0.5$, and $\lambda_{\min}=0.5$. It took the PSO solver 0.299 s to optimize the placement after 76 iterations. The results were $d_{T}=38.206$ mm and $\theta_{T}=-4.005$ deg. The optimized placement of the implanting tool is shown in Figure 17, with a YZ view (Figure 17a), an XY view (Figure 17b), an XZ view (Figure 17c), and an isometric view (Figure 17d).

**Figure 18:**
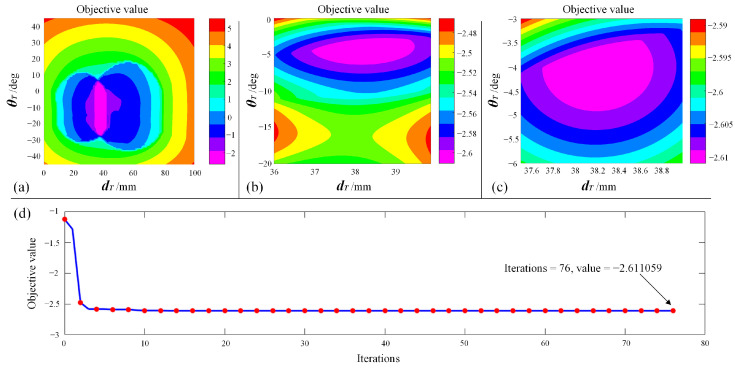
Verify the solution: (**a**) Global view of objective function. (**b**) Magnified view. (**c**) Local magnified view near the optimal value. (**d**) Convergence plot of PSO iterations.

To further verify whether the solution provided by the PSO solver is a desirable one, the solution space was traversed, generating contour plots of the objective function. A global view of the objective function is shown in Figure 18a, and a local magnified view of the region near the optimal solution is shown in Figure 18b,c. The convergence plot of the PSO iteration process is shown in Figure 18d. It can be seen from Figure 18 that the optimal solution is localized in the pink region, which corresponds to the solution where $d_{T}=38.206$ mm and $\theta_{T}=-4.005$ deg. As for the objective value, the optimized objective value is −2.611059, corresponding to the value of the pink region. With the contour plot, the solution obtained from the PSO solver is verified, meaning that this combination of $d_{T}$ and $\theta_{T}$ is a desirable one.

### 5.3. Discussion

The above-mentioned tests, including the FPSM and placement optimization, verify the feasibility and effectiveness of the proposed framework. In the framework, the FPSM provides a fast and intuitive spatial constraint, and the PSO solver can quickly find a target position/orientation for preoperative feeding and positioning.

In the establishment of the FPSM, it took 33.613 s to deal with the first stage of optimization when 15 slices were involved whose number of control points was 20. On average, it took the C++ solver 2.241 s to finish a slice, which is a bit slower than the MATLAB version and deviates from the usual expectations. This is because this part was mainly programmed using matrices operations for which MATLAB is usually one of the fastest environments. Even so, the time consumption can be completely accepted, given that the stereo scanning of the oral cavity usually lasts several or more minutes. Only half a minute of additional time for calculating an effective real-time collision detecting model is worthwhile. Moreover, with multi-core parallel calculation, in which different slices will be optimized using different computer cores, the time consumption will be much less and the solving speed will be accelerated by one or more times.

For the FPSM, by testing the coverage rate and consumed time, one can learn that the two performance indicators are in conflict: better coverage means poorer time consumption, and better time consumption means poorer coverage. With the testing data, choosing the number of control points to be 15 can balance the two conflicting indicators. Also, the parameter selection should not be limited to the testing data, since the computing platforms differ from each other. If there is no strict speed requirement or the computing platform is advanced, the slice number and point number can be higher to present a better surrogate model. Contrariwise, the FPSM should be simplified; for example, the slice number and the point number can be reduced to 10, in which the time consumption is decreased to only 12.227 s.

With the FPSM, the PSO solution is much faster. Assuming that 30000 triangles are included in the mesh, 30 particles are involved in PSO, and 100 iterations are taken for solving, there are almost 100000000 times of intersection tests between a triangle and the surgical tool for the entire PSO solving process. Additionally, the safety estimation for the objective function may well be complex since too many geometric elements have to be processed. Moreover, in some cases, the triangular mesh should be shrunk or offset, for which other complex geometry processing algorithms should be developed. When the triangular mesh is replaced by the FPSM, all the mentioned concerns can be eliminated immediately, making the FPSM an essential part of the proposed preoperative planning framework.

In addition, the FPSM is not only a part of the preoperative planning framework but also a multifunctional model for other tasks. For example, in real-time automatic obstacle-avoidance trajectory generating, the triangular mesh cannot be directly adopted because too much calculation has to be dealt with. Alternatively, the FPSM provides a fast and accurate collision-detection model which is suitable for online path planning or trajectory generation. Furthermore, in teleoperation under master-slave control, the FPSM can act as a real-time collision-alarming mechanism, which can warn the operator when the current situation is dangerous. Therefore, the FPSM is far more than its usage on paper and can be expanded to a considerable extent.

On the other hand, in terms of the PSO solver, the objective value reduces steeply during the first 10 times of iteration. In later iterations, the reduction of the objective value is much smaller. In the PSO program, when the optimal value does not change after 40 consecutive iterations, it is considered that the iteration has converged and the solver is exited. Thus, in the 36th iteration, the PSO solver finds the final solution. The time consumption of the PSO solver is 0.299 s, which is adequate for automatic preoperative planning, and there is even no need to wait. Combining the 3D visualization and the contour plot, a position/orientation with a minimal objective value is found. When the instrument moves in the planned direction, the risk of colliding with the tissue will be much lower. Although the objective function (safety evaluation) can be defined by many different expressions, meaning that the solution is not necessarily the best one, the risk is still lower than most of the manually defined preoperative planning. Therefore, the PSO solver is a satisfactory solving method when the FPSM is already presented.

**Figure 19:**
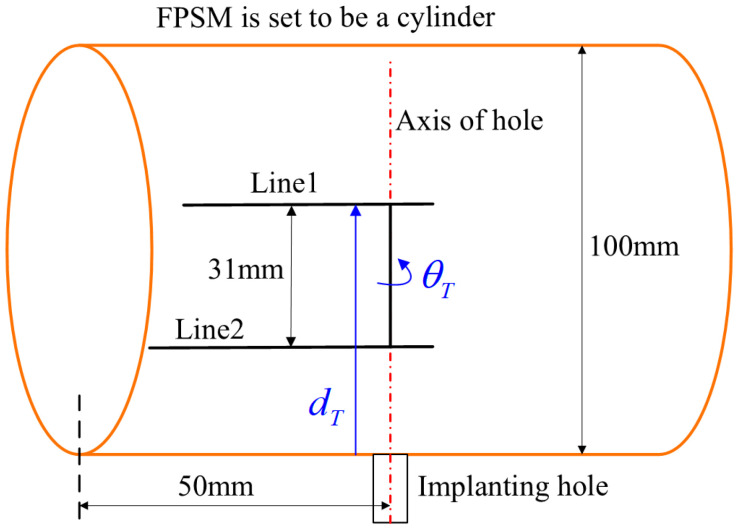
A simple example to compare objective functions in which FPSM is set to be a cylinder.

Additionally, for the PSO solver that is the second stage of optimization, to persuasively illustrate the effectiveness of the designed objective function (Equation (14)) against other common objective functions, another simple example can be adopted, as shown in Figure 19. In the example, a cylindrical FPSM whose radius is 50 mm is adopted, playing the role of an oral cavity without any complex geometry. The implanting target described in $\left\{Center\right\}$ is $\left(0,-50,50\right)$. The configurations and settings of the implanting instrument, VF, and the PSO parameters are all the same as the test in Figure 17. In Equation (14), the sum of the square root, whose expression is $k_{a}\sqrt{a}+k_{b}\sqrt{b}$, is chosen for building the optimizing objective. To make a comparison, two types of other objective functions whose expressions are $k_{a}a+k_{b}b$ and $k_{a}a^{2}+k_{b}b^{2}$ are introduced, respectively, written in Equations (21) and (22), which are two variants of Equation (14). The optimization results when Equation (14), Equation (21), and Equation (22) are, respectively, selected as the objective function are listed in Table 2.

(21) $$F_{\mathrm{safe}}=-\frac{\left(m_{1}+1\right)f_{\mathrm{safe}}^{\left(L1\right)}+\left(m_{2}+1\right)f_{\mathrm{safe}}^{\left(L2\right)}}{m_{1}+m_{2}+2}$$

(22) $$F_{\mathrm{safe}}=-\frac{\left(m_{1}+1\right){\left(f_{\mathrm{safe}}^{\left(L1\right)}\right)}^{2}+\left(m_{2}+1\right){\left(f_{\mathrm{safe}}^{\left(L2\right)}\right)}^{2}}{m_{1}+m_{2}+2}$$

**Figure 20:**
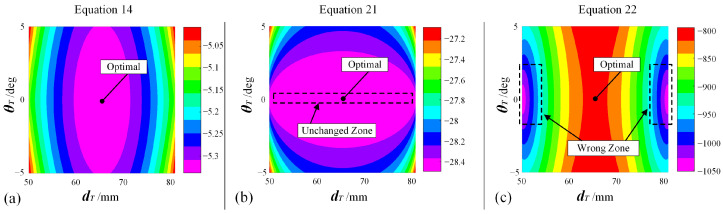
Contour plots for different variants of objective functions: (**a**) Equation (14), which is formally adopted as the objective function. (**b**) Equation (21), which is unacceptable because one DOF cannot be ensured to find the optimal value. (**c**) Equation (22), which is unacceptable because iteration will converge to wrong solutions.

From Figure 19, it can be easily drawn that the optimal solutions are $d_{T}=31+\left(100-31\right)/2=65.5$ and $\theta_{T}=0$. Among the different results in Table 2, using Equation (14) can obtain the optimal solution. Preliminarily, for Equation (21), whose type is $k_{a}a+k_{b}b$, the objective function value will remain unchanged when $\theta_{T}=0$, no matter how $d_{T}$ is adjusted. For Equation (22), whose type is $k_{a}a^{2}+k_{b}b^{2}$, it can be seen the result is the same, as line 2 coincides with the cylinder’s axis. Take this assumption that Equation (22) can be re-written as $d_{\mathrm{line}1}^{2}+d_{\mathrm{line}2}^{2}$ with $d_{line1}$, respectively, being the distances between line1/line2 and the cylinder. If $\theta_{T}=0$, $d_{line1}+d_{line2}=69$ will hold true if $d_{\mathrm{line}1}\leq$50 and $d_{\mathrm{line}2}\leq$ 50. Based on Equation (22), the larger $d_{\mathrm{line}1}^{2}+d_{\mathrm{line}2}^{2}$ is, the better the objective function. Following this, the optimal solution will never be $d_{\mathrm{line}1}=34.5$ and $d_{\mathrm{line}2}=34.5$, corresponding to the correct result driven by Equation (14). More deeply, contour plots were drawn, respectively, for Equations (14), (21), and (22), as shown in Figure 20, from which the unchanged zone of Equation (21) and the wrong zone of Equation (22) can be found, illustrating the correctness of the design for Equation (14). Therefore, the effectiveness of the objective function is preliminarily validated.

In summary, the preoperative framework can help the dentist determine how to place the implanting instrument in robot-assisted dental implant surgery, providing a faster but safer solution. The framework can be especially useful for master-slave operations or teleoperations. In teleoperation, the target for feeding should be preoperatively defined, and the moving process should be regulated by the virtual fixture to guarantee safety. With this framework, the target and the virtual fixture can be automatically determined without any participation by the operator. Moreover, in many rural areas and less developed countries, professional dentists are often under severe deficiency. This condition makes master-slave operations or teleoperations extremely important in case the patient is on one side of the world while the dentist is operating on another side of the world. The framework proposed in this paper can considerably enhance safety since teleoperation often lacks on-site feeling and sophisticated skill. When the target and virtual fixture can be automatically generated, such safety concerns will no longer be an obvious challenge. Hence, this work is also an essential expansion of the previous proposed study.

## 6. Conclusions and Future Work

This paper introduces an automatic preoperative planning framework to place the target of surgical instruments for feeding tasks in robot-assisted dental implant surgery. The framework is conducted through a two-stage optimization process, during which a finite parameter surrogate model is established and the instrument’s placement is solved. Conclusions can be listed as follows:

1. A new finite parameter surrogate model for the oral cavity, also called the FPSM, is designed using an array of closed curves in polar coordinates. After pre-assignment of the triangular mesh of the oral cavity, the model is solved during the first stage of optimization through gradient descent and penalty function methods.
2. The placement for the implanting instrument is solved using the PSO algorithm in the second stage of optimization, during which the FPSM serves as a safety estimator to drive the iteration to converge to the optimal solution.
3. The FPSM solver, the PSO solver, and a 3D visualization software are developed, and the performance of the entire framework is tested, preliminarily verifying the effectiveness of the proposed framework.
4. The FPSM has the feature of high real-time quality, accuracy, flexibility, and multifunctionality, which has great potential to play a role in preoperative planning, online trajectory planning, intraoperative motion regulating, and collision alarming. Combined with the PSO solver, the framework is an important expansion of the previously proposed work.

In the future, three works are planned which can further improve this study.

1. The FPSM will be used for other tasks and applications, including preoperative planning, online trajectory planning, intraoperative motion regulating, and collision alarming, which can bring more safety for other modes of surgical operation.
2. The proposed framework will be seamlessly integrated with master-salve control, virtual fixture, vision system, and system hardware. When all the modules are combined, a complete master-slave teleoperation test can be conducted, during which some control aspects including some non-linearities will be investigated and addressed.
3. Some artificial intelligence models, such as objective encoding and predicting networks, that are being studied in mobile robot navigation can be adopted and applied to the aspect of traditional robotic manipulators, which can bring more methods or approaches for robot-assisted oral surgery.

## Figures and Tables

**Table 1:** Coverage rate and consumed time for optimizing one slice.

*N*	Coverage (%)	Time (s)	*N*	Coverage (%)	Time (s)
3	59.24	0.171	12	90.30	1.161
4	71.09	0.262	13	87.29	1.247
5	60.14	0.215	14	91.28	1.255
6	82.31	0.303	15	88.23	1.926
7	79.11	0.346	16	91.42	1.860
8	79.98	0.459	17	92.05	3.098
9	82.63	0.406	18	90.92	3.969
10	84.07	0.665	19	93.17	4.867
11	86.24	0.653	20	90.87	4.911

**Table 2:** Optimization results when different variants of objective function are used.

Objective Function	$d_{T}/\mathrm{mm}$	$\theta_{T}/\deg$
Equation (14)	65.500	0.000
Equation (21)	55.304	0.000
Equation (22)	81.000	0.000

## Data Availability

Not applicable.
